# Serum ferritin predicted prognosis in patients with nasopharyngeal carcinoma

**DOI:** 10.1038/s41598-024-54627-3

**Published:** 2024-02-21

**Authors:** Zetan Chen, Zhongguo Liang, Kaihua Chen, Shuai Zhang, Xiaopeng Huang, Gang Wu, Xiaodong Zhu

**Affiliations:** 1 Department of Radiation Oncology, Guangxi Medical University Cancer Hospital, Nanning, 530021 Guangxi China; 2 Department of Radiation Oncology, Hainan General Hospital, Hainan Affiliated Hospital of Hainan Medical University, Haikou, 570311 Hainan China; 3 Department of Oncology, Wuming Hospital of Guangxi Medical University, Nanning, 530199 Guangxi China; 4 Key Laboratory of Early Prevention and Treatment for Regional High-Incidence-Tumor, Guangxi Medical University, Ministry of Education, Nanning, 530021 Guangxi China

**Keywords:** Cancer, Biomarkers, Medical research, Oncology, Risk factors

## Abstract

Elevated serum ferritin (SF) levels have been associated with poor prognosis in various cancer types, but its impact on nasopharyngeal carcinoma (NPC) remains unclear. This retrospective study analyzed clinical data from 252 non-metastatic NPC patients admitted to Hainan General Hospital between January 2014 and May 2016. SF levels were measured using the chemiluminescence method. Patients were categorized into low, medium, and high-level SF groups based on tertile median SF levels. Survival outcomes were assessed using Kaplan–Meier analysis and Cox regression models. The overall survival rates of the entire patient cohort at 1, 3, 5, and 8 years were 95.2%, 85.7%, 76.2%, and 68.9% respectively. The high-level SF group (SF > 164.00 ng/mL) had significantly worse overall survival (83.1 vs 96.3 months, P = 0.023) and progression-free survival (77.8 vs 93.3 months, P = 0.019) compared to the low-level SF group. Univariate and multivariate analyses confirmed that high SF levels, along with T3/T4 staging and N3 staging, were independent risk factors for poor prognosis. In conclusion, high SF levels are associated with shorter overall survival and progression-free survival in NPC patients.

## Introduction

Nasopharyngeal carcinoma (NPC) is one of the prevalent malignant tumors in southern China, particularly ranking as the sixth among all malignant tumors in Guangdong, Guangxi, and Hainan provinces. In high-risk areas, the annual male incidence rate can reach up to 27.2 per 100,000 individuals^1,2^. Early-stage NPC is commonly treated primarily with radiotherapy, while locally advanced NPC often requires a combination approach involving radiotherapy, chemotherapy, targeted therapy, and immunotherapy. Reported data indicate a 5-year overall survival (OS) rate of approximately 80% and a 5-year progression-free survival (PFS) rate of around 70% in patients with NPC^3,4^. Prognostic factors for NPC include tumor staging, Epstein–Barr virus DNA load, extracapsular spread of metastatic lymph nodes, serum lactate dehydrogenase levels, hemoglobin levels, nutritional indices, and serum ferritin (SF) levels^5–7^.

Ferritin, as an important iron-binding protein, plays a crucial role in regulating iron transportation and storage within cells. Recent studies have demonstrated the important role of SF in the growth, progression, and metastasis of various tumors. The impact of SF had been explored in other cancers^8–10^. It was rarely explored in NPC. And the precise mechanisms and clinical applications of SF in NPC remain controversial and inadequate. Previous studies have commonly set abnormal SF levels as above 215–300 ng/mL, considering levels higher than 215 ng/mL or even 300 ng/mL as abnormal^11^. However, clinical practice has indicated that NPC patients with SF levels exceeding 300 ng/mL are relatively rare. Therefore, this study aimed to collect clinical data from 252 patients with NPC and analyze their SF levels. By comparing the relationships between low, medium, and high SF levels and overall survival time and time to recurrence/metastasis in NPC patients, we observed that the low-level SF group had longer OS and PFS compared to the high-level SF group. This finding provides important insights for further exploring the mechanisms of SF in NPC and guiding the prognosis and clinical treatment of NPC.

## Materials and methods

### Patients

Retrospective collection of clinical data from non-metastatic NPC patients who received intensity-modulated radiotherapy at Hainan General Hospital from January 2014 to May 2016. All patients were restaged according to the 8th edition of the American Joint Committee on Cancer (AJCC) tumor staging criteria. Inclusion criteria were as follows: (1) pathologically diagnosed with nasopharyngeal carcinoma. (2) SF level measurement before initial treatment. (3) Staged as I–IVa (AJCC 8th edition). (4) Karnofsky Performance Score (KPS) ≥ 70. Patient information including gender, age, date of diagnosis, pathological type, hemoglobin level, serum ferritin level, treatment modality, and follow-up results were collected through the electronic medical record system and telephone interviews.

### Detection method of SF and patient grouping

For newly diagnosed non-metastatic NPC patients, before any treatment, 3 mL of fasting venous blood collected using an anticoagulant tube after at least 8 h of fasting in the morning. The blood samples were centrifuged at 1500–3000 rpm for 5–10 min to separate the serum, which was then tested within 1 h. Samples tested within 7 days were stored in a refrigerator at 2–8 °C. A protein chip was prepared by immobilizing monoclonal antibodies against SF on the membrane of the chip. The protein chip selectively captures ferritin antigens in the patient's serum, followed by the addition of a second antibody labeled with an enzyme tracer. The enzyme-catalyzed reaction generates a light signal, which is quantitatively measured using the HD-2001A biochip reader. The biochip reader and protein chip C12 were purchased from Huzhou Shukang Biological Technology Co., Ltd. A total of 252 NPC patients completed the testing and met the inclusion criteria. The SF test results of all 252 NPC patients were sorted from low to high, with the first 1–84 patients defined as the low-level SF group, the 85–168 patients as the medium-level SF group, and the 169–252 patients as the high-level SF group.

### Treatment and follow-up

Before treatment, all NPC patients underwent Nasopharyngoscope and biopsy, as well as MRI scans of the nasopharynx and neck (both plain and enhanced), chest CT, abdominal CT or ultrasound, and bone ECT to determine the TNM staging. Patients with stage I or II were primarily treated with intensity-modulated radiotherapy alone, while those with stage III or IVa received combined treatment with radiotherapy and chemotherapy. The radiotherapy regimen consisted of intensity-modulated radiotherapy using 6-MV X-rays, with a total dose of 68–71.04 Gy/30–32 F for the planning target volume (PTVnx) of the gross tumor volume (GTVnx) of the nasopharynx, 64–68 Gy/30–32 F for the PTV of the neck lymph nodes (PTVnd), 60–62 Gy/30–32 F for PTV1, and 54–56 Gy/30–32 F for PTV2. Radiotherapy was administered once daily for five consecutive days per week. For neoadjuvant chemotherapy, the TP regimen (docetaxel or paclitaxel plus cisplatin or nedaplatin or lobaplatin) or PF regimen (cisplatin or nedaplatin or lobaplatin plus 5-fluorouracil) was mainly used. Concurrent chemotherapy primarily consisted of a single agent platinum-based regimen (cisplatin or nedaplatin or lobaplatin) or the PF regimen. Chemotherapy was administered once every 3 weeks. Follow-up evaluations typically occurred one month after the completion of radiotherapy, with subsequent evaluations conducted every three months within the first 2 years after treatment, every six months between years 3 and 5, and annually after 5 years. The latest follow-up was conducted on June 10, 2023.

### Statistical analysis

Categorical variables were presented as frequencies and percentages. Continuous variables were reported as mean ± standard deviation (x ± s). The comparison of inter-group rates was conducted using the chi-square test. The Kaplan–Meier method was used to analyze the dynamic relationship between OS and PFS and follow-up time for all patients and various groups, and the log-rank test was utilized to compare the survival rates among low, medium, and high level SF groups. The factors that showed statistical significance in the univariate Cox analysis were included in the multivariate Cox regression analysis for survival analysis in order to identify independent prognostic factors for NPC patients. The statistical analyses were conducted using IBM SPSS 26.0 software. P < 0.05 indicated statistical significance for differences.

### Ethical approval

This study was approved by the Medical Ethics Committee of Hainan General Hospital (Ethical approval No.: Med-Eth-Re [2023] 315), and written informed consent was obtained from all patients. This study conformed to the relevant laws and regulations.

## Results

### Patient characteristics and treatment outcomes

**Figure 1:**
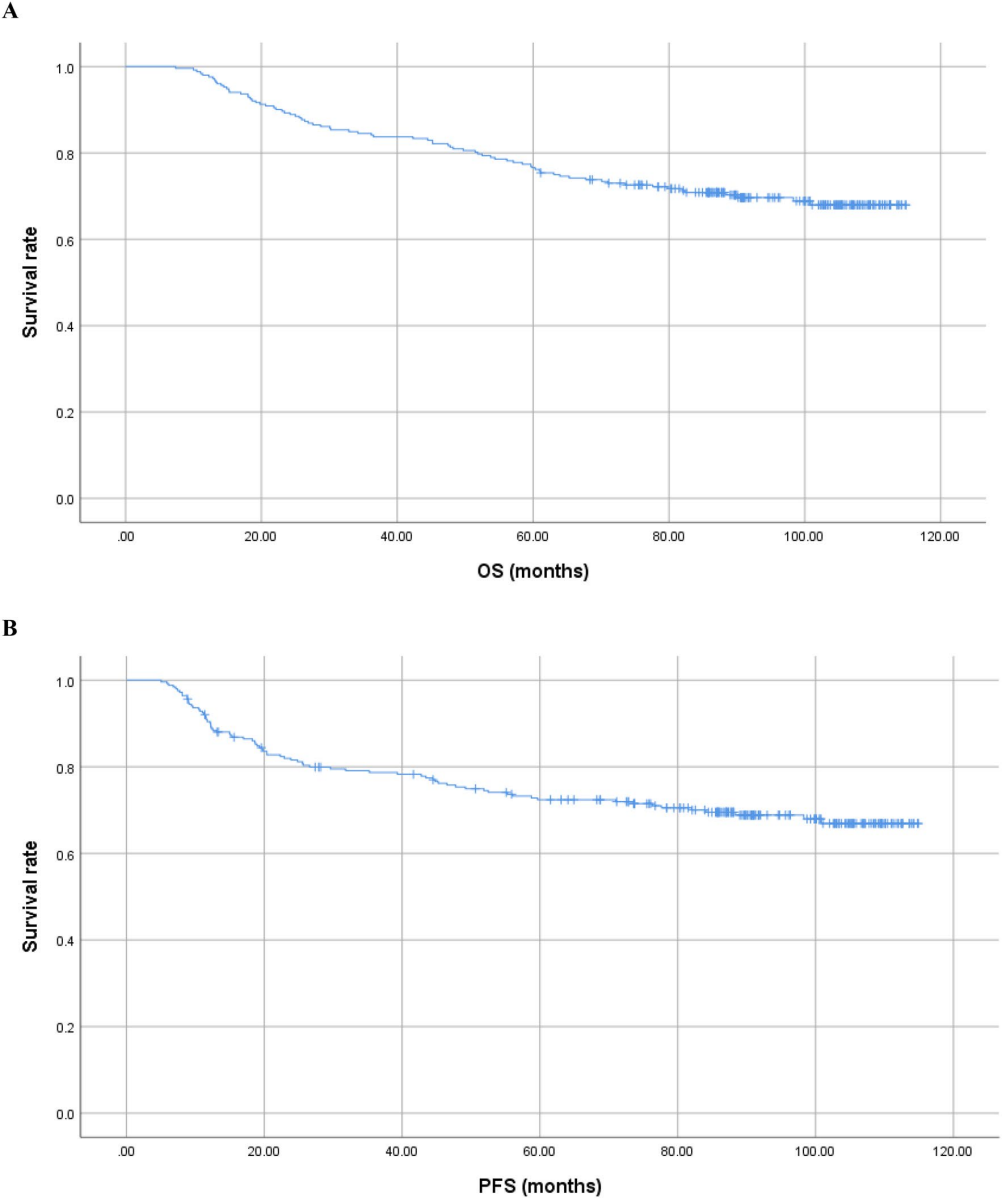
The OS and PFS of 252 patients with NPC. (**A**) The OS of 252 patients with NPC. (**B**) The PFS of 252 patients with NPC.

A total of 252 patients were included in this study, with a median follow-up period of 96.33 months (range 7.37–114.90 months). The clinical characteristics of all patients are described in Table 1. The male-to-female ratio in the entire group was 177:75 (2.36:1), with a median age of 49 years (range 19–80). The histopathology of 99.2% of patients was non-keratinizing carcinoma. The proportions of patients in stage III and IVa were 87.7% (stage III: 52.4%, stage IVa: 35.3%). The OS of the entire group ranged from 7.37 to 114.90 months, with a mean of (79.22 ± 30.72) months. The PFS ranged from 5.03 to 114.90 months, with a mean of (71.20 ± 36.00) months. The 1, 3, 5, and 8-year OS rates for the entire group were 95.2%, 85.7%, 76.2%, and 68.9%, respectively. The 1, 3, 5, and 8-year PFS rates for the entire group were 90.0%, 78.7%, 72.4%, and 67.9%, respectively. Please refer to Table 1 and Fig. 1 for more details.

**Table 1:** Baseline characteristics of 252 patients.

Characteristics	No. of cases	%
Gender		
Male	175	69.4
Female	77	30.6
Age (years)		
≤ 45	95	37.7
> 45	157	62.3
Pathology (WHO 2022)		
Keratinizing squamous cell carcinoma	2	0.8
Non-keratinizing carcinoma	250	99.2
Basaloid squamous cell carcinoma	0	0.0
T stage (the 8th AJCC)		
1	22	8.7
2	103	40.9
3	85	33.7
4	42	16.7
N stage (the 8th AJCC)		
0	9	3.6
1	64	25.4
2	126	50.0
3	53	21.0
Clinical TNM-stage (the 8th AJCC)		
I	3	1.2
II	28	11.1
III	132	52.4
IVa	89	35.3
IMRT prescribed dose		
GTVnx	68–71.04 Gy/30–32 F	
GTVnd	64–68 Gy/30–32 F	
Cycles of chemotherapy		
< 4	187	74.2
≥ 4	65	25.8
Hb (g/L)		
< 120	27	10.7
≥ 120	225	89.3
Serum ferritin (ng/mL)		
Low level group: 3.09–81.64	84	33.3
Medium level group: 81.73–164.00	84	33.3
High level group: 164.92–534.16	84	33.3

*AJCC* American joint committee on cancer*, IMRT* intensity-modulated radiotherapy*, GTVnx* gross tumor volume of the nasopharynx*, GTVnd* gross tumor volume of the neck lymph nodes*, Hb* hemoglobin*, WHO* world health organization, *AJCC* American joint committee on cancer.

### SF was an independent prognostic indicator of NPC

Based on the analysis of the tertile median value, patients with SF levels > 164 ng/mL were classified as the high-level SF group, while patients with SF levels ≤ 81.64 ng/mL were classified as the low-level SF group. Patients with SF levels between these two values were classified as the medium-level SF group. According to the SF assay kit instructions, only 15 patients had abnormal values, defined as SF ≥ 322 ng/mL, accounting for 5.95% of the entire patient population.

**Figure 2:**
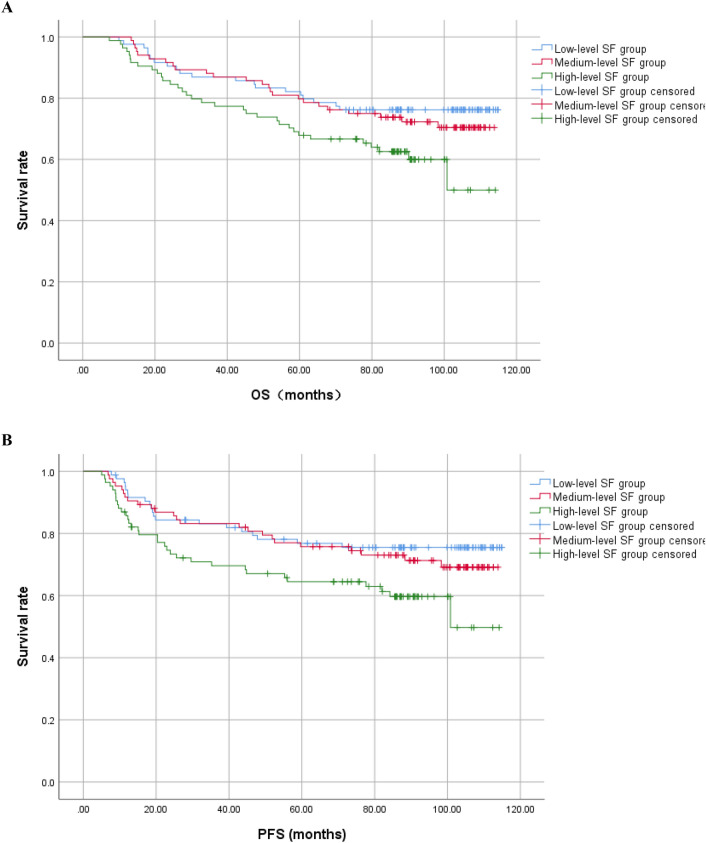
Survival time of low, medium and high SF levels groups. (**A**) The OS of the low, medium and high-level SF groups. (**B**) The PFS of the low, medium and high-level SF groups.

Survival analysis showed that the high-level SF group had significantly lower mean OS compared to the low-level SF group (83.1 vs 96.3 months, P = 0.023). The high-level SF group also had significantly PFS compared to the low-level ferritin group (77.8 vs 93.3 months, P = 0.019). The survival curves of patients in the low, medium, and high SF groups are presented in Fig. 2. The probabilities of 1, 3, and 5-year OS for the high-level ferritin group versus the low-level ferritin group were 95.2% (95% CI 90.6–99.9) versus 97.6% (95% CI 94.3–100.9), 77.4% (95% CI 68.2–86.5) versus 86.9% (95% CI 79.5–94.3), and 67.9% (95% CI 58.7–77.3) versus 81.0% (95% CI 73.2–88.8), with P values of 0.406, 0.107, and 0.035, respectively. The probabilities of 1, 3, and 5-year PFS for the high-level ferritin group versus the low-level ferritin group were 84.5% (95% CI 76.9–91.4) versus 92.8% (95% CI 87.9–98.1), 69.6% (95% CI 60.9–79.1) versus 81.9% (95% CI 74.3–89.7), and 64.5% (95% CI 54.9–73.9) versus 76.8% (95% CI 68.6–85.4), with P values of 0.049, 0.047, and 0.049, respectively.

Univariate Cox regression analysis showed that the high-level SF group (SF > 164.00 ng/mL) had a 1.900-fold increased risk of death compared to the low-level SF group (95% CI 1.088–3.319), and this difference was statistically significant (P = 0.024). The high-level SF group also had a 1.945-fold increased risk of recurrence or metastasis compared to the low-level SF group (95% CI 1.114–3.396), and this difference was statistically significant (P = 0.019). The medium-level SF group (SF: 81.73–164.00 ng/mL) had a 1.197-fold increased risk of death (95% CI 0.661–2.167) and a 1.205-fold increased risk of recurrence or metastasis (95% CI 0.666–2.182) compared to the low-level SF group, but these differences were not statistically significant (P > 0.05). T3/T4 stage, N3 stage, III/IVa clinical stage, and high SF levels were all significantly associated with OS in NPC patients (P = 0.010, P = 0.000, P = 0.040, P = 0.024). T3/T4 stage, N3 stage, III/IVa clinical stage, and high SF levels were also found to be significantly associated with PFS in NPC patients (P = 0.008, P = 0.000, P = 0.036, P = 0.019). Age, gender, hemoglobin level, and chemotherapy cycles were not found to be related to prognosis in NPC patients. See Tables 2 and 3 for details.

**Table 2:** Cox regression model to evaluate OS based on patient characteristics analyzed according to a univariable and multivariable analyses.

Variables	Univariate analysis			Multivariate analysis		
	HR	95% CI	P	HR	95% CI	P
Gender						
Female vs male	1.017	0.624–1.657	0.947			
Age (years)						
> 45 vs ≤ 45	0.759	0.471–1.224	0.258			
T stage						
3, 4 vs 1, 2	1.832	1.155–2.906	0.010	1.882	1.182–2.996	0.008
N stage						
3 vs 0, 1, 2	2.420	1.514–3.867	0.000	2.598	1.618–4.172	0.000
Clinical TNM-stage						
III, IVa vs I, II	2.872	1.050–7.860	0.040			
Cycles of chemotherapy						
≥ 4 vs < 4	1.042	0.626–1.734	0.875			
Hb (g/L)						
≥ 120 vs < 120	1.200	0.552–2.610	0.646			
Serum ferritin (ng/mL)						
Medium level group vs low level group	1.197	0.661–2.167	0.553	1.244	0.682–2.270	0.476
High level group vs low level group	1.900	1.088–3.319	0.024	1.915	1.093–3.357	0.023

*OS* overall survival*, HR* hazard ratio*, CI* confidence interval*, Hb* hemoglobin.

**Table 3:** Cox regression model to evaluate PFS based on patient characteristics analyzed according to a univariable and multivariable analyses.

Variables	Univariate analysis			Multivariate analysis		
	HR	95% CI	P	HR	95% CI	P
Gender						
Female vs male	0.984	0.604–1.603	0.949			
Age (years)						
> 45 vs ≤ 45	1.295	0.803–2.087	0.289			
T stage						
3, 4 vs 1, 2	1.873	1.181–2.971	0.008	1.963	1.231–3.131	0.005
N stage						
3 vs 0, 1, 2	2.799	1.749–4.478	0.000	3.193	1.978–5.155	0.000
Clinical TNM-stage						
III, IVa vs I, II	2.931	1.071–8.021	0.036			
Cycles of chemotherapy						
≥ 4 vs < 4	1.086	0.652–1.808	0.751			
Hb (g/L)						
≥ 120 vs < 120	1.192	0.548–2.593	0.658			
Serum ferritin (ng/mL)						
Medium level group vs low level group	1.205	0.666–2.182	0.538	1.328	0.725–2.432	0.358
High level group vs low level group	1.945	1.114–3.396	0.019	2.068	1.177–3.632	0.011

*PFS* progression-free survival*, HR* hazard ratio*, CI* confidence interval*, Hb* hemoglobin.

Multivariate Cox regression analysis results showed that T3/T4 stage, N3 stage, and high SF levels were independent factors affecting OS in NPC patients (P = 0.008, P = 0.000, P = 0.023). Additionally, T3/T4 stage, N3 stage, and high SF levels were identified as independent factors influencing PFS in NPC patients (P = 0.005, P = 0.000, P = 0.011). See Tables 2 and 3 for details.

## Discussion

Due to the limited treatment response and unfavorable prognosis after recurrence and metastasis of nasopharyngeal carcinoma (NPC), the median survival period is reported to be only 22.1 months^12^. Therefore, identifying indicators that can early reflect the risk of recurrence and metastasis in NPC holds significant value.

Literature review reveals that elevated SF levels are associated with poor prognosis in various tumors such as lung cancer, breast cancer, multiple myeloma, pancreatic cancer, and others^8,9,13,14^. However, the relationship between SF levels and prognosis in NPC remains controversial. An earlier study suggested that increased SF levels are associated with advanced staging of head and neck cancer^15^. In 1996, a study conducted in a Hong Kong hospital measured SF levels in 184 healthy individuals, taking the mean + 2 standard deviations (535 ng/mL) as the cutoff value for abnormal ferritin levels. During the same period, SF levels were also measured in 279 patients with untreated NPC, and it was found that 37 patients had SF levels ≥ 535 ng/mL. Follow-up revealed that the proportion of patients with high SF levels who experienced metastasis within 1 year (34.2%) was significantly higher than that of patients with SF levels < 535 ng/mL (10.3%), indicating a close association between high SF levels and NPC metastasis^16^. Conversely, a study in 2004 investigating the relationship between SF levels and prognosis in 160 patients with NPC found no difference in outcome between the high SF group and the low SF group, with the median value of 144 ng/mL as the cutoff^17^. In our present study, we found the following results: firstly, high SF levels before treatment (the top one-third of ferritin levels in the entire patient group) were highly correlated with OS and PFS in patients with stage I–IVa NPC, with higher SF levels associated with shorter OS and PFS. Secondly, univariate and multivariate Cox analyses revealed that elevated SF levels were an independent prognostic indicator for stage I–IVa NPC. Our findings contrasted with the study in 2004, which could possibly be attributed to the smaller sample size and the use of 2-dimensional radiation therapy in the earlier study, as well as the differences in cutoff values. In our study, the cutoff value for the high-level SF group was > 164 ng/mL. However, studies conducted by a team of researchers involving 503 patients and 622 patients with non-metastatic NPC who underwent intensity-modulated radiotherapy, demonstrated consistent results with our study^7^. These studies found that SF levels above 292 μg/L and 300 μg/L, respectively, were independent prognostic indicators for NPC.

It is generally believed that elevated SF levels may indicate increased iron load or other causes, while decreased SF levels are typically indicative of iron deficiency. Ferritin in tissues is typically glycosylated, whereas SF, which circulates in the blood, is mostly non-glycosylated and carries the light chain L subunit, not actively involved in iron transport^11,18^. Elevated SF levels can be associated with chronic inflammation, infectious diseases, alcoholic or non-alcoholic liver diseases, autoimmune disorders, and tumors, among other factors. Although the exact source, secretion pathway, and physiological/pathological roles of SF remain unclear, its elevation is often used as a nonspecific tumor marker due to its association with various types of cancer.

Ferritin, discovered by French scientist in 1937, is a hollow nanocage capable of binding up to 4500 iron atoms and is responsible for iron storage and release. Ferritin is composed of 24 subunits, including a heavy chain H subunit (FHC, FTH) or a light chain L subunit^19^. Ferritin carrying FHC subunit is mostly derived from the heart or red blood cells, while ferritin carrying the light chain L subunit is mostly derived from the liver and spleen. FHC exhibits enzymatic activity and can convert ferrous iron to ferric iron. In the human body, normal ferrous iron, once absorbed in the small intestine, typically exists in a protein-bound form, including ferritin, which stores oxidized iron as ferric iron. Only a small amount of iron remains semi-free in the cytoplasm, known as the labile iron pool, which serves as a buffer in normal cells to maintain iron balance and cellular homeostasis, allowing cells to function normally. In cancer cells or cells with pathological changes, the labile iron pool is increased due to the accelerated cell proliferation and increased demand for iron. The increased free iron can react with hydrogen peroxide, resulting in the Fenton reaction (Fe^2+^  + H_2_O_2_ → Fe^3+^  + ^**·**^OH + OH^−^) and other chemical reactions, generating oxygen-derived free radicals^20^. This in turn damages cells, leading to genetic mutations, cell death, or cellular transformation. Studies have shown a close correlation between FHC and tumor occurrence and progression, including cell proliferation, growth inhibition, immune evasion, suppression of cancer cell death, and angiogenesis^21^. In non-small cell lung cancer cell lines, FHC functions as a tumor suppressor gene by relieving the suppression of p53 transcription mediated by miR-125b. This leads to increased expression of the pro-apoptotic protein BAX and inhibition of the anti-apoptotic protein Bcl-2, ultimately inducing tumor cell apoptosis^22^. Similarly, silencing the FHC gene increases ROS levels in breast cancer and lung cancer cells, activating the CXCR4/CXCL12 pathway, resulting in the acquisition of an epithelial-mesenchymal transition phenotype and promoting proliferation and migration ability^21^.

Interestingly, ferritin is associated with radiation sensitivity. Accumulation of lipid droplets has been observed in radioresistant cells of breast cancer, lung cancer, bladder cancer, gliomas, and prostate cancer. The accumulation of lipid droplets releases specific lipids that prevent iron-dependent cell death, and upregulates the expression of ferritin. Silencing the ferritin heavy chain gene significantly reduces the accumulation of lipid droplets in the above-mentioned cell lines and increases radiation sensitivity, indicating the involvement of ferritin in the formation of radioresistance in tumor cells^23^. In this study, all NPC patients received intensity-modulated radiation therapy. The development of radiation resistance may be a possible cause of treatment failure (recurrence or metastasis). Patients with high serum ferritin levels had a lower PFS and a higher probability of treatment failure, suggesting a correlation with the ferritin-mediated radiation resistance mechanism. However, as this study was a single-center retrospective study, no definitive causal relationship can be established. External validation and more basic and clinical research are expected to confirm these findings.

In current clinical practice, risk assessment for recurrence and metastasis in nasopharyngeal cancer is based on high-risk factors, including advanced stage (T4 and/or N3), pre-treatment EBV-DNA > 4000 copies/mL, and post-treatment failure to normalize EBV-DNA levels. Oral capecitabine/S-1 adjuvant chemotherapy has been shown to prolong patient survival^4,24^. The use of serum ferritin biomarker in conjunction with serological tests offers advantages such as rapid, convenient, and simple operation. Therefore, further multicenter studies are necessary to determine the cut-off value of serum ferritin for predicting recurrence and metastasis in nasopharyngeal cancer, and to apply it in clinical practice.

In summary, SF levels were found to be associated with the prognosis of NPC. This study revealed that non-metastatic NPC patients in the high-level SF group (SF ≥ 164.00 ng/mL) had significantly shorter OS and PFS compared to the low-level SF group. These findings indicate that SF has a certain clinical value in the assessment of prognosis for NPC. However, further investigations are required to elucidate the precise mechanisms by which SF influences the development of NPC.

## Data Availability

All the data used and analyzed in this study can be obtained from the corresponding author.
